# Production of bacterial cellulose using *Gluconacetobacter kombuchae* immobilized on *Luffa aegyptiaca* support

**DOI:** 10.1038/s41598-021-82596-4

**Published:** 2021-02-03

**Authors:** Sameeha Syed Abdul Rahman, T. Vaishnavi, G. Sai Vidyasri, K. Sathya, P. Priyanka, Ponnusami Venkatachalam, Sugumaran Karuppiah

**Affiliations:** Bioprocess Engineering Laboratory, Centre for Bioenergy, School of Chemical & Biotechnology, SASTRA Deemed To Be University, Thanjavur, Tamil Nadu 613401 India

**Keywords:** Biotechnology, Materials science

## Abstract

The present work report for the first time on the production of bacterial cellulose (BC) using natural loofa sponge (*Luffa aegyptiaca*) as a scaffold for the immobilization of *Gluconacetobacter kombuchae*. Bacterial cellulose (BC) are recently gained more attention in several fields including biological and biomedical applications due to their outstanding physico-chemical characteristics including high thermal stability, easy biodegradability, good water holding capacity, high tensile strength, and high degree of polymerization. The increase in requirement of alternative method for the enhancement of BC production under economical aspect develops a positive impact in large scale industries. In this study, *Luffa aegyptiaca* (LA) was introduced in a separate fermentation medium so as to enhance the concentration of BC production by *Gluconacetobacter kombuchae*. Different process/medium parameters such as initial pH, static/shaking condition, inoculum size, nitrogen source, C/N ratio, supplements (ethanol and acetic acid) were analysed for the production of bacterial cellulose using LA support. The maximum yield of BC was obtained using following condition: culturing condition -shaking; initial pH − 5.5; nitrogen source- yeast extract, C/N ratio – 40 and supplement—ethanol. The characterization of the BC was examined using Fourier Transform Infra-Red spectroscopy and thermo gravimetric analysis. The biofilm formation on the surface of LA was examined by SEM photographs. Thus, implementation of LA as a support in shaking fermentation under suitable medium/process variables enhanced the BC production.

## Introduction

Bacterial cellulose (BC), one among the carbohydrate polymers, has been increasing applications in the past decades owing to its outstanding characteristics such as hydrophilicity, biocompatibility, crystallinity, tensile strength, moldability, degree of polymerization, biodegradability and thermal stability^1–6^. It is widely used in numerous commercial applications, some of which are: (i) preparation of artificial skin, artificial blood vessel, nano structured biomaterials in tissue engineering, (ii) synthesis of biodegradable materials, wound cleansing material in biomedical field; (iii) manufacturing of low-calorific desserts, fabricated foods and salads in food industry, (iv) as a stability enhancer in paper industry and (v) cosmetics^7–9^. Unlike plant cellulose, BC produced through microbial fermentation has high purity and does not require any energy consuming steps such as hydrolysis and delignification due to the absence of lignin, pectin and hemicellulose^10^. BC has both amorphous and crystalline structure and the crystallinity degree is significantly influenced by the culturing condition, medium formulation and nature of raw material used^11,12^.

Although it is structurally similar with plant cellulose (β- 1,4-glucans, (C_6_H_12_O_6_)_n_), certain bacteria, specifically acetic acid bacteria (AAB) like *Agrobacterium, Aerobacterium, Rhizobium, Salmonella, Sarcina, Azetobacter, Achromobacter, Komagataeibacter* has the capability of producing Bacterial cellulose (BC) by their metabolic activity^13^. The preferable carbon sources for the commercial production of BC are glucose, fructose and glycerol ^14^. The suitable medium formulation is essential for supporting the growth of microorganism and facilitating the production of BC^15^.

The production of BC has been significantly enhanced by the optimizing medium variables (carbon, nitrogen, potassium, magnesium, phosphorus and sulfur), culturing conditions (pH, temperature, static culture, shaking culture) and submerged/solid state fermentation^16–20^. The culturing conditions could affect the formation of BC and its morphological structures including mat like structure or pellicle (static) and pellets or granules (shaking). Several researchers demonstrated that the shaking culture condition is more preferable for the production of BC over static culture condition due to the requirement of minimum floor area, mixing and lesser incubation time^15,21–23^. However, it is difficult to achieve more yield of BC using such AABs producing strains under shaking condition. To overcome the difficulties in shaking culture and increase the fermentation efficiency, numerous attempts have been made to improve the production of BC: (i) using genetically modified AAB; (ii) screening of essential medium components; (iii) optimizing culturing conditions/parameters; (iv) modifying the configuration or type of reactors such as cylindrical silicone membrane vessel, rotating disk bioreactor, immobilized cell reactor, hollow fiber reactor and biofilm reactor^24–30^. Due to better nutrient transport, high biomass density, high volumetric productivity and also lower capital cost requirement, BC production with immobilized cell cultivation (biofilm on the matrix) are preferable over conventional cultivation^31^. Biofilm formation takes place during fermentation, where the microorganism gets attached over the surface of the support naturally by microbial immobilization technique^32^. Several researchers reported the production of ethanol, lactic acid, nisin, succinic acid, bacteriocin, BC and pullulan using biofilm reactors to enhance the productivity^31,33–38^. In the present study, the production of BC was investigated using immobilized *Gluconacetobacter kombuchae* on *Luffa aegyptiaca* (LA), as a novel and cost-effective biomass support.

LA is a genus of tropical and sub-tropical plant in the cucumber family of *Cucurbitaceae*. This plant is generally grown in countries like India, Japan, China and Africa. The chemical composition of LA has protein (4.2%), lipid (1.08%), ash (1.04%), fiber (55.78%) and carbohydrate (37.81%)^39^. Luffa fiber contains cellulose (60%), hemi-cellulose (30%) and lignin (10%)^40^. Luffa species have been employed as a potential carrier for microbial/plant cell/animal cell immobilization due to low biodegradability, appropriate ratio of lignin & cellulose, structural stability, large space for cell growth, fixed cells with high cell density, rich composition of lignin, cellulose high mass transfer efficiency in fixed bed, wide range pH stability, exposure to high temperature for repeated sterilization, reusability, texture, shape and high porosity^41^. It has been successfully used as a carrier/scaffold material for microbial cultivation, plant cell cultivation and biomedical engineering filed^42–45^. *Luffa* species has the following physico-chemical properties in Table 1.

**Table 1:** Physico-chemical properties of Luffa species.

Property	Value
Porosity (%)	79–93^41,46^
Density (g/cc)	0.02–0.92^41,46^
Specific volume (cc/g)	21–29^41^
Specific pore volume (cc/g)	31 ± 6^46^
Pore size (µm	335 ± 65^46^
Fiber diameter (mm)	0.63 ± 0.22^46^
Young's modulus (MPa)	918–1897^47^
Tensile strength (MPa)	53.82–91.63^47^
Elongation (%)	5.16–9.81^47^
Moisture regain	7.1–10.4^47^
Relative crystallinity (%)	24.4–47.1^47^
Degradation point (°C)	269.6–324.6^47^

**Figure 1:**
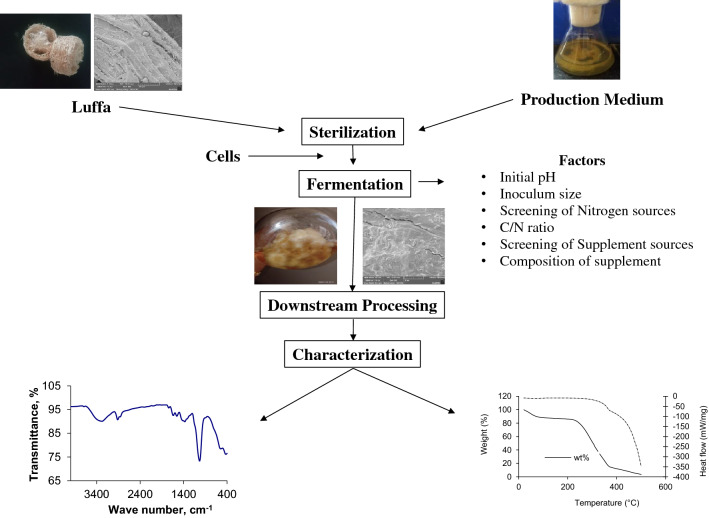
Schematic diagram for the production of BC using immobilized *Gluconacetobacter kombuchae* on LA.

The objective of this work was to examine the implementation of immobilization support, *Luffa aegyptiaca* for BC production by *GluconAcetobacter kombuchae*, which was divided in to three specific objectives: (i) comparison of BC production with/without immobilized matrix under shaking/static condition and also to analyze the material property of *Luffa aegyptiaca* scanning electron microscopy analysis (SEM); (ii) effects of different factors namely initial pH, inoculum size, nitrogen source, C/N ratio, supplement on BC production using suitable condition. (iii) to analyze the thermal & structural properties of produced BC thermogravimetric (TGA) & FTIR analysis respectively. Although, several plastic supports have been employed in biosynthesis of BC in previous studies (Cheng et al. 2009, 2010), this study pioneers at using *Luffa aegyptiaca* as a novel support for BC synthesis. To goodness of our knowledge, this is the first report that describes the production of bacterial cellulose using LA as a scaffold for the immobilization of *Gluconacetobacter kombuchae*. The overall schematic representation for the production of BC is described in Fig. 1.

## Result and discussion

### Identification of initial pH and culturing conditions on BC production

**Figure 2:**
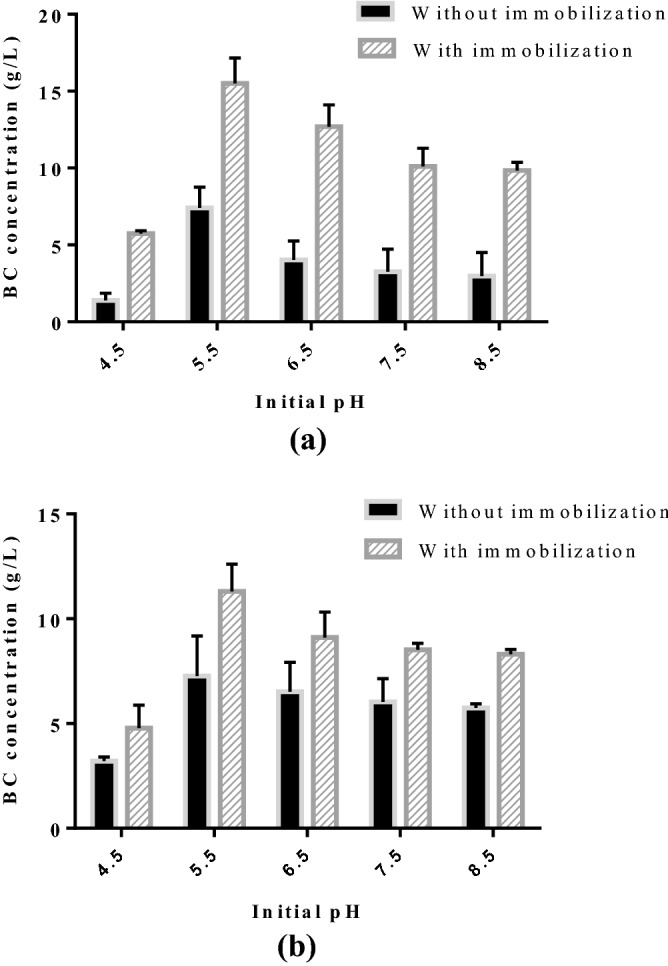
Identification of culturing condition and initial pH in the production of BC (**a**) shaking condition; (**b**) static condition (Fermentation time = 15 days).

The medium pH is an essential parameter affecting the activity of primary enzymes responsible for BC production, thereby should be optimized in order to achieve maximum concentration of BC. Therefore, the medium pH was varied over the range (4.5–8.5) using HCl or NaOH before sterilization^48^. Figure 2 explains the effect of intial pH culturing condition on the production of BC using free cell and immobilized cells. The fermentation was carried out using free cells and immobilized cells under shaking and static cultivation for 15 d. Under shaking condition, fermentation medium containing immobilized cells and free cells resulted maximum BC concentration of 15.5 ± 1.65 g/L and 7.4 ± 1.34 g/L respectively (Fig. 2a). In static condition, the maximum BC concentration of 11.3 ± 1.31 g/L and 7.27 ± 1.91 g/L were obtained at pH 5.5 for immobilized cells and free cells respectively (Fig. 2b). From experimental data, it is evident that the production of BC was favored by immobilized cells with shaking. The production of BC in shaking condition was about 1.3 times more than that in static culturing conditions under optimum initial pH. The static culture for BC production has several disadvantages than shaking cultivation due to the lack of essential nutrients in the aerobic region, lowere volumetric productivity and low oxygen level required for microbial growth in the static condition^49,50^.

A low and high pH resulted in low concentration of BC under shaking and static cultivation using free cells and immobilized cells. This could be attributed to the decrease in the activity of key enzymes responsible for BC production. Previous studies have reported that maximum BC was produced at an initial pH of 5 by *Leifsonia sp.* CBNU-EW3 (4 g/L) and *K. europaeus* SGP37 (6.07 ± 0.40 g/L)^51,52^. Santoso et al., 2020 demonstrated the maximum BC production of 3.906 g/L with optimum pH of 5.4 under static condition using sucrose medium^53^. Similarly, several researchers have illustrated the maximum BC production at an initial pH of 5 in static condition^48,51,52^. Rangaswamy et al., 2015 identified the optimum pH of 6 for maximizing BC production (2.1 g/L) by *Gluconacetobacter sp.* RV28 isolated from rotten fruits. Several authors have reported that BC production was more suitable when the medium pH was ranging from 5 to 6^54,55^. Thus, BC production using immobilized cell on LA support was higher than that in the absence of support. At initial pH 5.5 and shaking condition, the maximum BC production of 15.5 ± 1.65 g/L was achieved using immobilized cell with LA support, which was approximately about two times more than that of the absence of the support.

### Culturing condition

**Figure 3:**
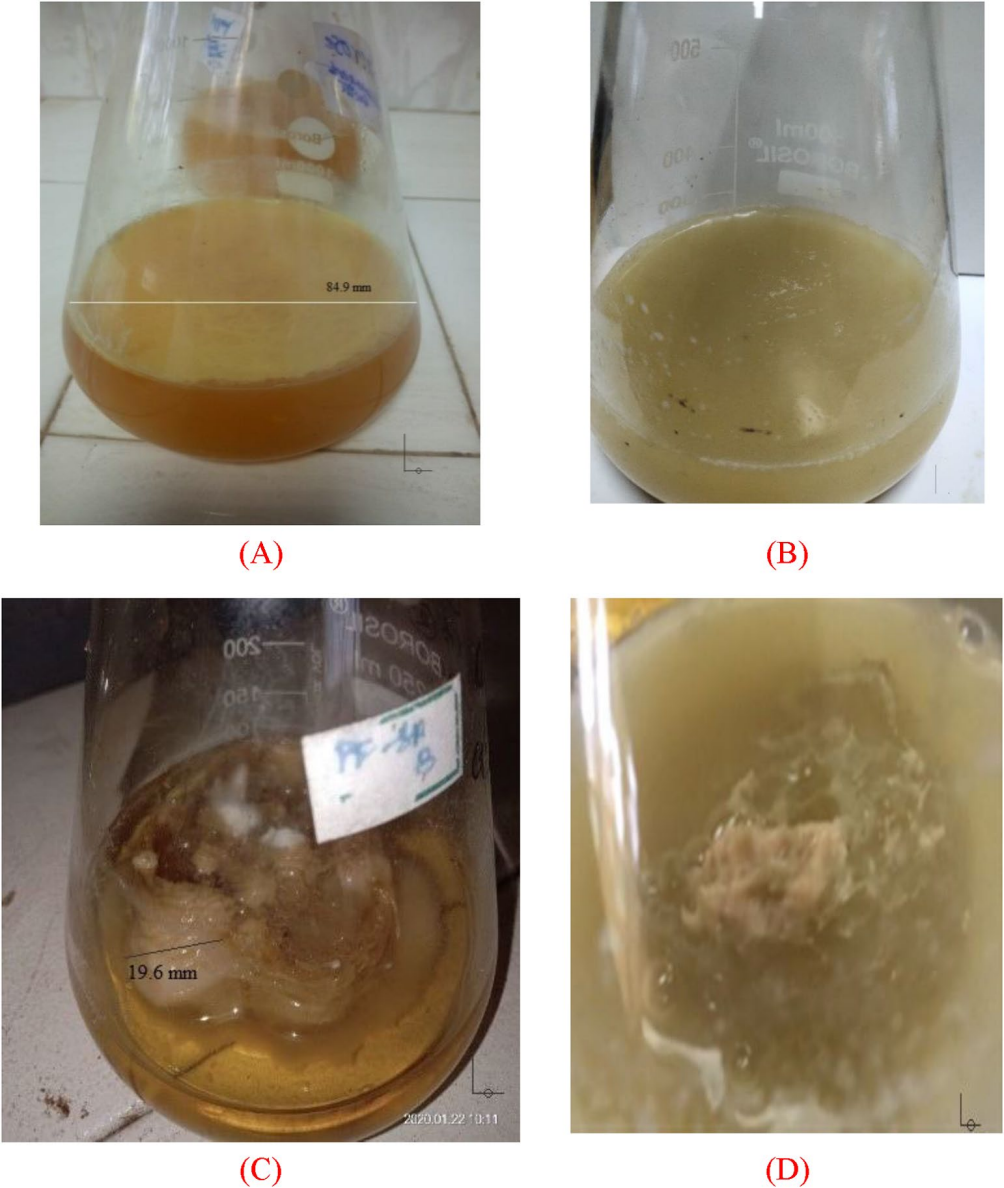
Photgraphic images for BC formation (**a**) static condition without LA matrix; (**b**) shaking condition without LA matrix; (**c**) static condition with LA matrix; (**d**) shaking condition with LA matrix.

Culturing condition (static/shaking) is an essential parameter for the production of BC with various forms. The various forms namely fibrous suspension, pellets, spheres or irregular masses were observed under shaking condition. The produced BC is not restricting the transport of essential nutrients for microbial growth at the air–liquid interface^49,56^. Tanskul et al. observed the various forms of BC such as granular /tapioca-pearls/few irregular shapes and interconnected reticular pellicles under shaking and static condition respectively^57^. The different forms of BC synthesis were observed due to the disruptive effect of strong mechanical means (aeration/shaking) on the formation of hydrogen bonds within BC chain^58^. The photographic images for BC formation on with and without LA support (culture medium 150 ml) are shown in Fig. 3. Figure 3A depicts the formation of BC mat on the top surface of the production medium under static condtion without LA support. This observation was consistent with previous findings (Heieh et al., 2016). Under shaking condition without LA support, viscous and turbid nature of culture was observed as shown in Fig. 3B. This might be due to the uniform elaboration of produced BC throughout the medium. Whereas with LA support, irregular BC aggrgates are formaed on the surface of LA support as shown in Fig. 3C,D under both static and shaking cultivation.

### Surface morphology of *Luffa ageyptiaca*

**Figure 4:**
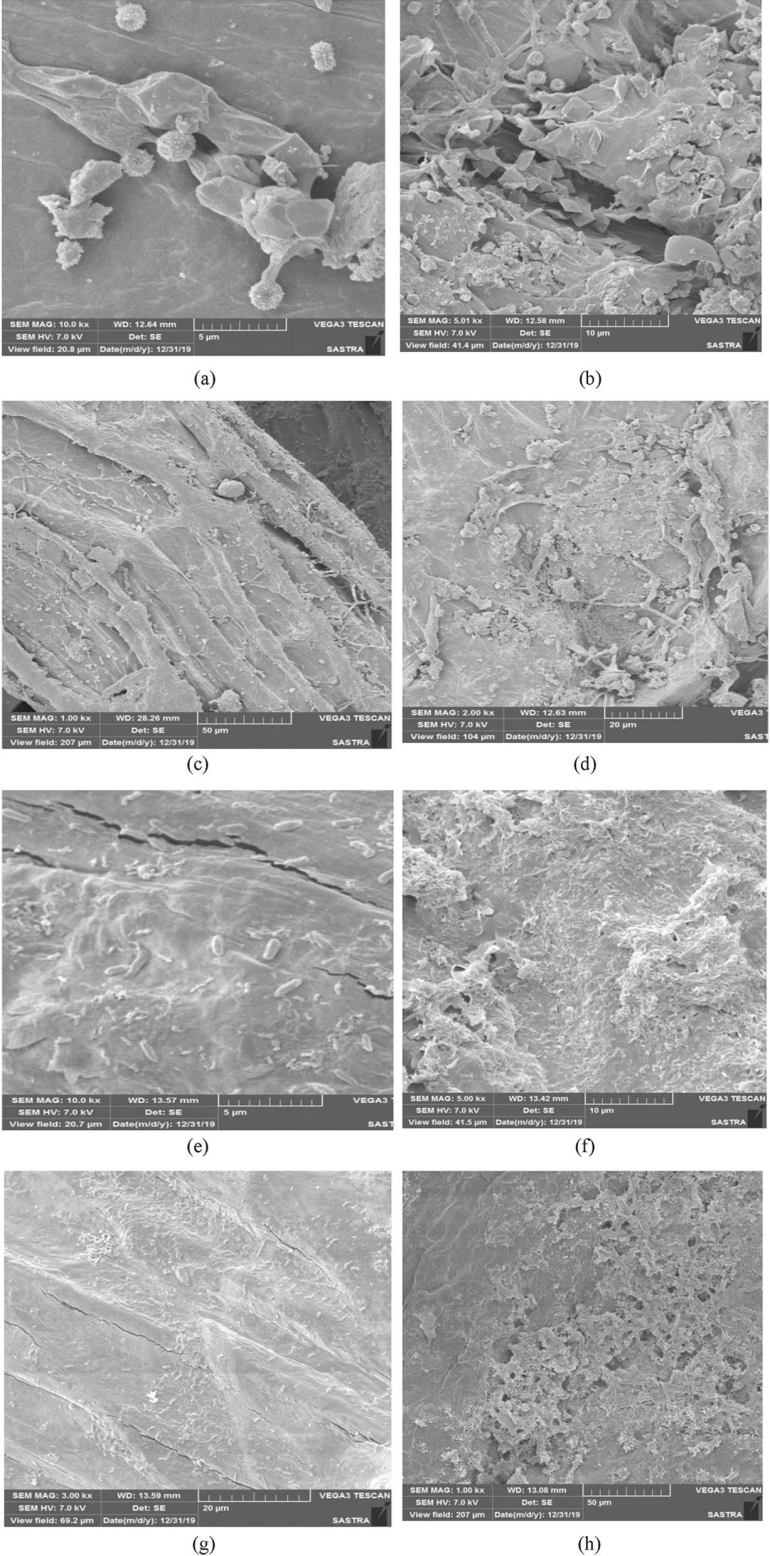
Surface morphology of *Luffa ageyptiaca* (**a**–**d**) before cells attachment; (**e**–**h**) After cells attachment.

SEM analysis is used to evaluate the surface morphological changes of *Luffa ageyptiaca* during the production of BC. The SEM photographs of *Luffa ageyptiaca* before and after fermentation were illustrated in Fig. 4a–h with different magnification. The SEM micrographs of *Luffa ageyptiaca* before fermentation show dense, rough, cavities and highly heterogeneous surface with different shape and size as shown in Fig. 4a–d. This kind of surface texture is essential for attachment of cells. During fermentation, *Gluconacetobacter kombuchae* cells were growing predominantly over the surface of *Luffa ageyptiaca* with suitable fermentation condition and medium formulation for the production of BC*.* The rod-shaped cells were clearly visible over the surface of the *Luffa ageyptiaca* in different magnification (3 kx and 10 kx) as shown in Fig. 4e–h during fermentation. The morphological texture of *Luffa ageyptiaca* in terms of rough, cavities, pores and highly heterogeneous surface has significantly changed due to the attachment of cells and accumulation of BC over *Luffa ageyptiaca* during fermentation.

### Inoculum size

**Figure 5:**
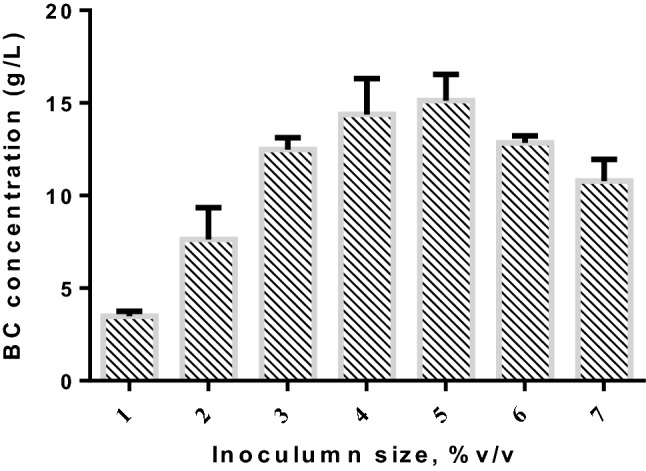
Effect of inoculum size on the production of BC using immobilized cells in biofilm fermentation (Fermentation time = 15 day; initial pH = 5.5; shaking condition).

Microbial polysaccharide production is significantly influenced by inoculum size in batch cultivation^59^. The larger inoculum size with active seed culture minimizes the length of adaptation (lag period) phase and facilitates the biomass concentration with a short fermentation time leading to higher production of exopolysaccharides^60^. Figure 5 shows that the effect of inoculum size ranging from 1 to 7% (v/v) on the concentration of BC using immobilized cells with initial pH 5.5 for 15 days incubation. The result illustrated that the concentration of BC raised significantly by increasing the size of inoculum from 1 to 5% v/v and then decreased. The maximum BC concentration of 15.12 ± 1.42 g L^−1^ was obtained with an inoculum size of 5% (v/v) after 15 days of fermentation than 4%, v/v (14.38 ± 1.94 g L^−1^). The significant decrease in BC concentration was noted above 5% (v/v) of inoculum size. The possible reason might be that the rapid consumption of feed stock responsible for BC production at larger inoculum size^61^. And also, the quick utilization of the limiting substrate in the early stage of batch fermentation with larger inoculum size above the optimum, was not suitable for the elaboration of BC in the final stage of fermentation^62^. In the larger inoculum size containing a greater number of active bacterial cells above the certain level might limit too much composition of oxygen in the production medium. The larger number of microorganisms had been killed due to the insufficient composition of oxygen in the aerobic batch fermentation. However, if the inoculum size goes below a certain level, the number of bacterial cells in fermentation medium responsible for BC elaboration was minimum, and hence the efficiency of fermentation was low^62^. Therefore, the produced BC might be subjected to the category of product formation directly coupled with energy metabolism and also a growth associated product^63^. Similarly, the maximum production was achieved under shaking condition using SH medium containing glucose with 5% (v/v) of inoculum size^64^. Hornung et al., 2006 reported that a significant number of young cells in the aerobic fermentation medium is required for the significant production of BC^65^. Thus, according to the previous findings, it might be expected from this present study, that 5% (v/v) cell density of *Gluconacetobacter kombuchae* MTCC (6913) facilitated the maximum BC production in the aerobic medium.

### Screening of nitrogen source

**Figure 6:**
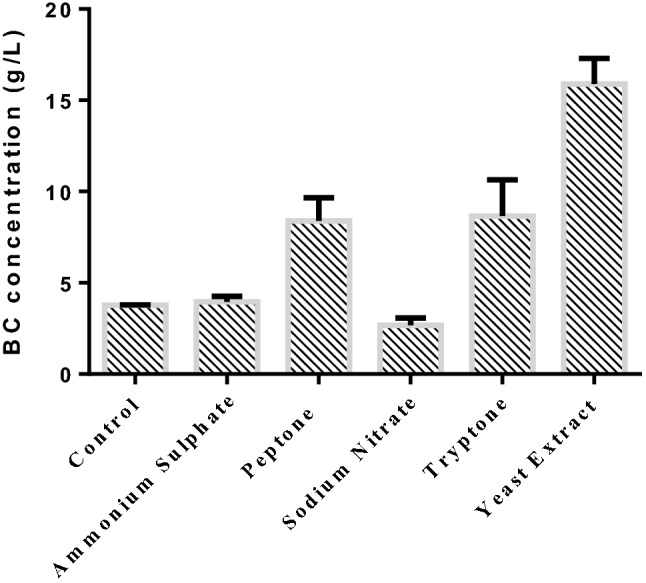
Screening of nitrogen source on the production of BC using immobilized cells with initial pH 5.5 under shaking condition in biofilm fermentation (inoculum size: 5%, v/v, fermentation time: 15 days; initial pH 5.5).

Nitrogen source is essential for supporting the growth of cells. The presence of Nitrogen in the production medium might not be coupled with the biosynthesis of BC ^15,66^ in an industrial scale process. In order to keep the composition of nitrogen content in the production medium at desired level, various nitrogen sources namely ammonium sulphate, peptone, sodium nitrite, tryptone were considered and substituted with yeast extract (3 g/L) on the basis of equivalent nitrogen content. The comparison of N-sources for the production of BC was evaluated using immobilised cells at initial pH 5.5 under shaking condition for 15 days as shown in Fig. 6. Peptone and Tryptone in the medium resulted the BC concentration of 8.4 ± 1.27 g/L and 8.67 ± 1.98 g/L respectively. Ammonium sulphate and sodium nitrite showed lower BC concentration of 3.95 ± 0.3 and 2.67 ± 0.42 g/L respectively. It was noted that there was no significant difference in the concentration of BC between the control medium and ammonium sulphate or sodium nitrate in the production medium. Maximum BC concentration of 15.9 ± 1.4 g/L was obtained using yeast extract in the production medium among other N-sources used with equal composition of nitrogen content. The production of BC using yeast extract was approximately 2 folds higher than peptone or tryptone in the production medium. The reason might be that yeast extract has a rich source of nutrients including carbon, amino acids and vitamin B complex essential for for stimulating the cellular growth as well as the synthesis of product^67,68^. Similar to the findings, Embuscado et al., 1994 reported that inorganic N-sources such as sodium nitrate as well as ammonium sulphate resulted in relatively low production of BC during fermentation^69^.

### Effect of carbon to nitrogen ratio

**Figure 7:**
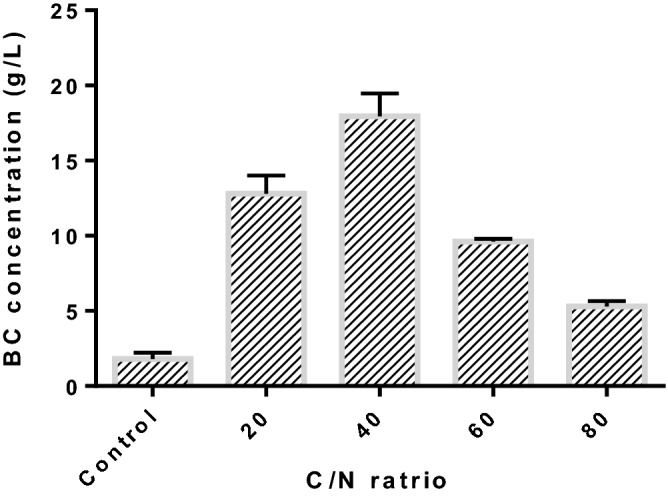
Effect of carbon to nitrogen ratio on BC production under shaking condition using biofilm fermentation (inoculum size: 5%, v/v, fermentation time: 15 d; initial pH: 5.5; nitrogen source: yeast extract).

The production of microbial polysaccharides is highly influenced by primary medium factor, C/N ratio, weight of carbon per weight of nitrogen). This factor could facilitate the metabolic processes resulting in polysaccharide elaboration from protein synthesis during fermentation^70,71^. As illustrated in Fig. 7, the effect of C/N ratio on the production of BC was investigated by adjusting the composition of carbon content, while keeping the same concentration of nitrogen in the production medium. When the C/N ratio was increased, BC concentration significantly increased till a C/N ratio of 40 and then decreased. A maximum BC concentration of 17.6 ± 1.52 g/L was achieved with C/N ratio of 40. It was noticed that relatively a very low concentration of BC (1.8 ± 0.42 g/L) was obtained in the absence of sucrose indicating the carbon limiting nature of polysaccharide. When the C/N ratio was increased as high as above 40, the production of BC drastically decreased due to the high concentration of limiting substrate (carbon source) leading to substrate inhibition. At high substrate concentration, fructose produced by the hydrolysis of sucrose might be acting as an osmotic stressor. In such situation, the presence of fermentable sugars in the production medium could not diffuse across the cellular membrane leading to trigger defense mechanisms against osmotic stress^72^. This effect was described by Seesuriyachan et al. for other xopolysaccharides^73^. The previous literature illustrated that high carbon to nitrogen ratio would provide relatively low yield of exopolysaccharide as well as cell density^74^.

### Effect of supplement on BC production

**Figure 8:**
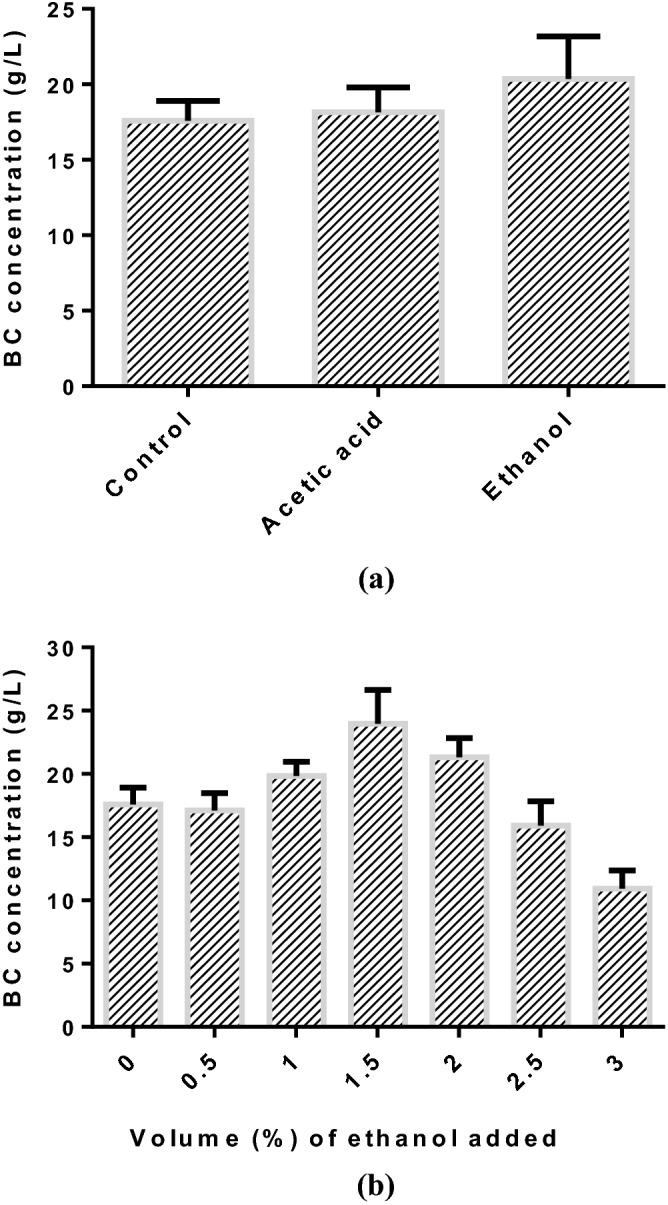
(**a**) screening of supplement; (**b**) effect of supplement concentration on BC concentration ((inoculum size: 5%, v/v, fermentation time: 15 d; initial pH = 5.5; Nitrogen source: Yeast extract; C/N ratio: 40).

The previous literature demonstrated that the secondary substrates or supplements (organic acids or ethanol) are essential to facilitate the production of BC^21,75–77^. Thus, in the present study, ethanol and acetic acid was used as supplement carbon source for BC production and their effect on BC production was investigated to identify the suitable secondary substrate (Fig. 8a). It was noted that production of BC (20.37 ± 2.81 g/L) was relatively higher using ethanol as a supplement in the production medium than acetic acid (18.16 ± 1.65 g/L). It was seen that the difference in the concentration of BC obtained between acetic acid and control was not significant. Even though, consumption of ethanol and acetic acid in the medium might suddenly provide energy source in the biosynthetic path way of TCA cycle due to the liberation of ATP, thus, facilitating the fermentation process for BC production^78^; the presence of acetic acid in the medium eventually decreased the pH leading to low concentration of BC. This might be due to the fact that the suitable pH range is 5–6 for the production of BC^54^. Ethanol in the production medium could be acting as a facilitator for the production of BC. Ethanol can produce the reduced form of NADH, which lowers the redox potential. Low redox potential is essential for the optimal production of BC^79^. Thus, ethanol was selected as a secondary substrate for the enhancement of BC production in the currentt study.

Figure 8b described the effect of ethanol concentration on the concentration of BC using immobilized cell biofilm reactor. To investigate its effect, different concentrations of ethanol (0–3%, v/v) were introduced in to the production medium. The concentration of BC was significantly increased to maximum level at 1.5%, v/v of ethanol concentration and then decreased. As shown in Fig. 8b, the supplement, ethanol with concentration at 1.5%, v/v significantly improved the BC production 23.97 ± 2.68 g/L as compared to control (17.59 ± 1.32 g/L) shown in Fig. 8a. It was noted that the production of BC slightly improved by 32% due to the addition of ethanol. And also, ethanol in the production medium has the ability of eliminating the non-cellulose producing strains during the shaking cultivation^15^. It is also noted that high concentrations of ethanol (above 1.5%, v/v) in the cuture medium caused reduction in BC concentration owing to the inhibitory effect on cell growth^77^.

In Table 2 the volumetric productivity of BC obtained using LA has been compared with the previous reported. The productivity obtained with LA was better or comparable than the previous reports. The improved producitivty is probably due to the following the reasons: (i) cultivation of high density of biomass on the surface of LA support; (ii) enough space for the cultivation of biomass; (iii) mess like structure for the better attachment of cells; (iv) re-usability of support (iv) facilitate the better micro-environmental condition for the continuous transport of essential nutrientsvolumetric productivity of BC^31^**.** Thus, LA appears to be a promising support for facilitating BC production.

**Table 2:** Comparison of produced BC using LA support with previous studies.

Carbon source	Volumetric productivity of BC (g L^−1^ day^−1^)	Condition	Microorganism
Waste beer yeast with sucrose (3%)	0.514	Static	*Gluconoacetobacter hansenii* CGMCC 3917^1^
Treated molasses (45.8 g/L)	1.8	Static	*Gluconoacetobacter intermedius* SNT-1^80^
Modified HS medium with glucose (15 g/L) and CSL (25 g/L)	0.963	Static	*Gluconoacetobacter hansenii*^81^
Schramm and Hestrin (glucose 20 g/L)	0.652 0.367 0.032	Static Shaking Stirred	*Gluconoacetobacter hansenii*^57^
Cheese whey	1.817	Static	*K. sucrofermentans* B-11267^82^
*Jerusalem artichoke* hydrolysate	0.75	Static	Immobilized *Komagataeibacter xylinum* B-12429 on polyvinylalcohol^83^
*Jerusalem artichoke* hydrolysate	0.42	Static	*Komagataeibacter xylinum* B-12429^83^
Corn steep liquor-fructose	1.41	Shaking	*Acetobacter xylinum* ATCC 700178; PCS Biofilm reactor^84^
Sucrose (25 g/L)	0.753 1.598	Static Shaking	Immobilized *Gluconoacetobacter kombuchae* MTCC6913 on *Luffa aegyptiaca* (In this study)

### Characterization of bacterial cellulose

#### Thermal stability and FTIR spectra

**Figure 9:**
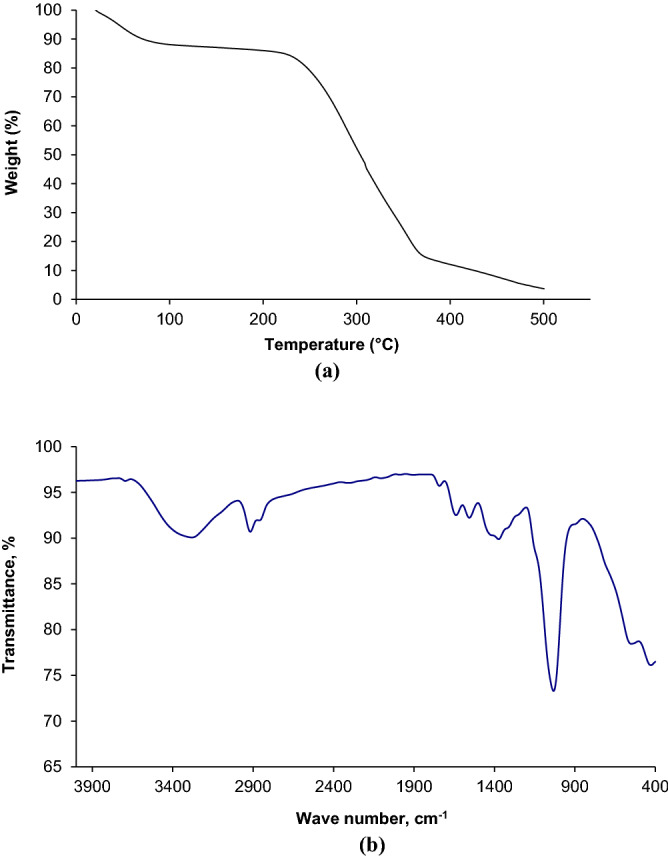
Characterization of produced BC (**a**) Thermal gravimetric analysis; (**b**) FTIR spectrum.

The thermal stability of microbial polysaccharide was examined by TGA curve for the determination of thermal transitions, melting point and decomposition point^85,86^. Thermo-gravimetric analysis (TGA) for produced BC by immobilized *Gluconacetobacter kombuchae* using sucrose medium is shown in Fig. 9a. The degree of crystallinity/amorphous, stability of functional groups and molecular mass of the microbial polysaccharide could greatly influence the thermal degradation^14,85^. Majorly three thermal degradation regions were observed in TG curve as shown in the Fig. 9a. A small variation (approximately 4%) in weight loss (89–228 °C) followed by around 68% of dynamic weight loss (228–370 °C) obtained in the second stage of thermal degradation. It might be due to the rapid thermal transformation of back bone structure of polysaccharide by breaking down the linkages of C–C, C–O. Above 370 °C , the final stage of thermal degradation accomplished by thermal decomposition of repeating monomer units followed by char formation^87^. The decomposition temperature of the produced BC using biofilm fermentation was found to be 270 °C.

FTIR spectrum for produced BC using biofilm fermentation is shown in Fig. 9b. The intensity of the transmittance peak around 1400 cm^−1^ has also been well associated with the degree of crystallinity, i.e., “crystallinity band”^88,89^. Due to the low intensity peak observed around 1400 cm^−1^ indicating the presence of crystallinity of BC, the absorption band at 879 cm^−1^ has been assigned to –C–O–C stretching at (1→4) glycosidic linkage indicating “amorphous” absorption band^90^. Based on the presence of characteristic band , it is noticed that the produced BC has both amorphous and crystalline nature^11,91^. The peaks at 1651, 1425, 1368 indicates H–O–H bending of absorbed water, CH_2_ scissoring, and CH bending^92,93^. Other features of polysaccharide were also found from the spectra. The characteristic bands of cellulose (type I) was confirmed due to the presence of stretching vibration of hydroxyl groups (–OH) at 3281.3 cm^−1^, the stretching vibration of (–CH–) at 2917.6 cm^−1^. The presence of absorption peak around 2800 cm^−1^, 1032 cm^−1^ and 1600 cm^−1^ were due to the symmetric stretching vibration of methyl (–CH_3_), stretching vibrations of the pyranose ring and stretching of -O-C-O functional group respectively^94^. The peak appeared at 1032, 896 and 554 cm^−1^ showed –C–O–H– bond of carbohydrates (or) –C–O–C– vibrational stretching in the pyranose ring, antisymmetric out of phase ring stretching of β-glucosidic linkage between glucose units and carbohydrate peak respectively^93,95,96^. The obtained results were compared with existing reports^97–100^.

## Materials

### Chemical reagents and medium components

The medium componets namely sucrose, yeast extract, beef extract, agar, tryptone and peptone and chemical reagents namely ammonium sulfate, sodium nitrate, magnesium sulphate, potassium sulphate, sodium hydroxide, acetic acid and ethanol were obtained from from Himedia, India with a purity standard more than 95%. The microbial strain, *Gluconacetobacter kombuchae* MTCC6913 was procured from the Microbial Type Culture Collection (MTCC) & Gene Bank, Chandigarh, India.

## Methods

**Figure 10:**
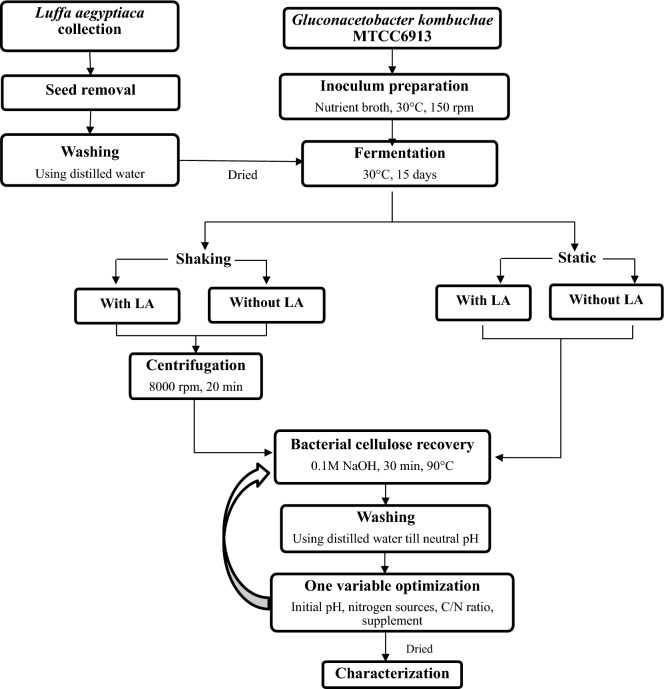
The schematic representation of methodology for Bacterial cellulose production.

The schematic representation of materials and method for the production of BC using LA support was depicted in Fig. 10.

### Microorganism and culture condion

The microorganism was cultured using nutrient agar medium containing the following composition (in g/L): yeast extract – 1.5, beef extract − 1.5; and agar − 15. The growth medium was prepared using nutrient broth, sterilized and cooled. Two loops of inoculum from nutrient agar culture were aseptically transferred into the nutrient broth (seed culture medium) and the culture was maintained in an orbital shaker at 30 °C and 150 rpm for one day. This seed culture was sub-cultured once in two weeks.

### Collection of the support material *Luffa aegyptiaca* and batch fermentation

*Luffa aegyptiaca* (LA) is a vegetative support material with mesh like structure. In the Loofah or sponge gourd (*Luffa aegyptiaca*), interior fibers made up of mesh like structure was used as a support for the attachment of cells and facilitate the biofilm formation during fermentation. LA was collected from local villages, Thanjavur, India. After removing the seeds, LA was thoroughly washed with distilled water to remove dirt and other impurities, dried in sun-light and then cut in to small pieces. The support was kept in 250 mL Erlenmeyer flask, in which the production medium (150 ml) was added in the following composition^101^ in g/L: sucrose − 25, yeast extract − 3, magnesium sulphate − 5, potassium sulphate—3 and pH 7.0 (before sterilization). The production medium with support material was sterilized at 121 °C for 20 min. After cooling, 5% v/v of inoculum (1.71 ± 0.07 × 10^7^ CFU/mL and OD _at 600 nm_ = 0.53) was introduced into the production medium. During fermentation, the cells as well as the pellicle were attached over the surface of support for facilitating the production of BC.

### Recovery of BC

After fermentation, BC was recovered according to the method prescribed by Cheng et al. 2009 with some modifications^37^. The medium was subjected to autoclaving at 121 °C to inactivate the cellular protein. The BC formed on the LA was carefully scraped using a knife and along with the fermentation broth, centrifuged at 8000 rpm for 20 min. The precipitated BC with cells was treated with 0.1 N NaOH for 30 min at 90 °C, for the removal of cells and other medium constituents. The sample was then washed with distilled water till the pH of wash water became neutral. It was oven-dried at 70 °C for 12 h to remove the moisture content in the BC pellet and then weighed. The concentration of BC was expressed in dry basis (gram of dry BC per liter of sample taken for analysis). All the experiments were investigated in triplicates and the data was represented by mean ± SD.

### Factors affecting the production of BC

The effect of initial pH (4.5–8.5) on the concentration of BC was studied in four ways according to the cell type and culturing condition: (i) shaking culture with immobilized cells on LA; (ii) shaking culture with suspended cells; (iii) static culture with immobilized cells on LA; (iv) static culture with suspended cells. Culturing condition, initial pH and the cell types that produced maximum BC were selected and used in further studies. The different factors namely inoculum size (1–14%, v/v), nitrogen source (ammonium sulfate, yeast extract, sodium nitrate, tryptone and peptone), carbon to nitrogen (C/N) ratio (0–80), supplement source (acetic acid and ethanol) and composition of supplement (0.5–3%, v/v) on concentration of BC were investigated in batch fermentation.

### Characterization of BC

With the help of Scanning Electron Microscope (VEGA3 XM, TESCAN USA, Inc.,), the surface morphology of LA and cells attached over LA (biofilm formation) were examined. The functional groups and thermal properties (TGA curve) of produced BC were examined by FTIR spectroscopy (PerkinElmer Spectrum Version 10.03.09) and thermogravimetric analysis (SDT Q600 V20.9 Build 20).

For FTIR analysis, the sample preparation was done by KBr pellet method. The sample spectrum was scanned from a range of 4000 cm^−1^ to 400 cm^−1^. In Thermo-gravimetric analysis, the sample was subjected to heating from 30 to 500 °C at a rate of 10 °C per min, under the atmosphere of nitrogen with a flow rate of 100 mL per min. The percentage lost by weight of the sample was evaluated as a function of temperature. Prior to thermal analysis, the sample to be analyzed was dried sufficiently enough in hot air oven to remove the moisture in the sample.

## Conclusion

The influence of LA support on the production of BC under static and shaking condition was examined. The heterogeneous surface morphology of LA interms of roughness, cavities and spiral structure facilitating the better attachment of cells improved BC production under shaking condition. The various morphological forms of BC was observed with/without LA support under static and shaking condtion. Based on the maximum yield of BC, shaking condition with LA support for cell attachment was considered for further studies. The suitable medium factors/process conditions for the microbial synthesis of BC were found to be: initial pH-5.5, inoculum size – 5% (v/v), nitrogen source – yeast extract, C/N ratio – 40; supplement – ethanol (1.5% v/v). Thermal and structural characterization of produced BC was investigated using TGA and FTIR analysis. Thermal decomposition of produced BC was found to be 270 °C. Based on the easy availability, cost effective and surface morphology of LA for the better attachment of cells yielding the maximum yield of BC, *Luffa ageyptiaca* could be a promising scaffold for bioprocess applications.
